# Ferroptosis as a Therapeutic Vulnerability to Overcome Chemoresistance in Gastric Cancer

**DOI:** 10.3390/ph19060949

**Published:** 2026-06-17

**Authors:** Carlo Calabrese, Tiziana Notarangelo

**Affiliations:** Laboratory of Preclinical and Translational Research, Centro di Riferimento Oncologico della Basilicata (IRCCS CROB), 85028 Rionero in Vulture, PZ, Italy

**Keywords:** ferroptosis, chemoresistance, lipid peroxidation, iron metabolism, targeted therapies

## Abstract

Gastric cancer (GC) remains a leading cause of cancer-related mortality worldwide, with treatment outcomes often severely constrained by the emergence of chemoresistance. Ferroptosis, a regulated, iron-dependent form of cell death driven by lethal lipid peroxidation, is a vulnerability that cancer cells actively evade to survive. Its dysregulation has been increasingly linked to therapeutic resistance across multiple malignancies, including GC. Dysregulation of the cystine–glutathione–GPX4 antioxidant system, iron metabolism, and lipid remodeling allows tumor cells to escape ferroptosis, but targeting ferroptosis represents a promising strategy to overcome therapeutic resistance and restore sensitivity to cancer treatments. This review discusses the molecular regulation of ferroptosis, its contribution to chemoresistance, and translational strategies to exploit ferroptosis in cancer therapy.

## 1. Introduction

Gastric cancer (GC) constitutes a major public health challenge worldwide, representing the fifth most frequently diagnosed cancer and the fourth leading cause of cancer-related death [1]. GC accounts for approximately 5.6% of all new cancer diagnoses and 7.7% of global cancer-related mortality [2,3].

Cisplatin is a platinum-based chemotherapeutic agent widely used as a first-line treatment for GC and other malignancies [4]. Its antitumor activity primarily relies on the formation of DNA crosslinks and the induction of reactive oxygen species (ROS), ultimately triggering apoptotic or non-apoptotic cell death pathways [5,6,7]. Despite advances in early diagnosis, surgical techniques, and systemic therapies, prognosis remains poor in advanced GC due to late diagnosis and the frequent emergence of therapeutic resistance [8]. Consequently, median overall survival in advanced GC often remains below 12 months, reflecting the aggressive nature of the disease and the adaptive capacity of tumor cells under therapeutic pressure [9].

Both intrinsic and acquired resistance mechanisms limit the clinical efficacy of cisplatin-based therapy. Tumor cells evade cisplatin-induced cytotoxicity through enhanced DNA repair, altered drug uptake or efflux, suppression of regulated cell death pathways, and activation of antioxidant defense systems. Among these, the nuclear factor erythroid 2-related factor 2 (NRF2) pathway plays a pivotal role by detoxifying ROS and mitigating oxidative damage, thereby promoting tumor cell survival under chemotherapeutic stress [7,10,11]. Recent evidence suggests that ferroptosis suppression represents a critical adaptive mechanism underlying platinum resistance in gastric cancer. Beyond its canonical DNA-damaging activity, cisplatin induces excessive intracellular ROS production, mitochondrial dysfunction, and oxidative stress, thereby creating a pro-ferroptotic environment characterized by lipid peroxidation and redox imbalance [12]. To survive under these conditions, cancer cells activate antioxidant pathways that counteract ferroptotic cell death. Among these, the SLC7A11–GSH–GPX4 axis acts as a major ferroptosis checkpoint by promoting cystine uptake, glutathione synthesis, and detoxification of phospholipid hydroperoxides, thus preventing lethal lipid peroxidation [13]. Increased expression of SLC7A11 and GPX4 has been associated with ferroptosis resistance and reduced sensitivity to platinum-based chemotherapy in several malignancies, including gastric cancer [13,14]. Furthermore, NRF2-mediated transcriptional programs regulate antioxidant defenses, glutathione metabolism, and iron homeostasis, thereby limiting ferroptotic susceptibility and facilitating adaptation to chemotherapy-induced oxidative stress [15]. Collectively, these findings indicate that ferroptosis evasion is not merely associated with chemoresistance but may constitute a fundamental survival strategy enabling gastric cancer cells to withstand cisplatin-induced oxidative damage. Among the drug resistance mechanisms, ferroptosis has emerged as a regulated form of cell death, mechanistically distinct from apoptosis or necroptosis. Ferroptosis is characterized by iron-dependent accumulation of lipid hydroperoxides, mitochondrial shrinkage, increased membrane density, and loss of cristae [16,17,18]. Increasing evidence highlights ferroptosis as a critical determinant of therapeutic response, tumor progression, and drug resistance in GC [19]. Dysregulated iron homeostasis, lipid metabolism, and antioxidant defenses collectively modulate ferroptosis susceptibility, positioning this pathway as a promising therapeutic target [20]. This review discusses the molecular regulation of ferroptosis, its role in chemoresistance, and emerging translational approaches to target ferroptosis in GC treatment.

## 2. Drug Resistance in GC

Chemotherapy resistance can be classified as primary (intrinsic) or secondary (acquired), depending on whether it arises before or after drug exposure [7,17,21]. Patients may be resistant to a single agent or, more critically, exhibit multidrug resistance (MDR), a phenotype associated with poor prognosis, aggressive tumor behavior, and cross-resistance to a broad range of drugs with diverse mechanisms of action [10]. MDR arises from multiple, often overlapping mechanisms, including enhanced drug inactivation and efflux, improved repair of chemotherapy-induced damage, activation of pro-survival signaling pathways, and disruption of cell death programs [5,11,22,23].

Among these, drug efflux plays a pivotal role. Aberrant expression of P-glycoprotein (P-gp), an ATP-binding cassette (ABC) transporter that extrudes numerous chemotherapeutic agents, and glutathione S-transferases (GSTs), which catalyze glutathione (GSH) conjugation reactions involved in cellular detoxification, critically contributes to drug resistance [24]. While P-gp mediates the efflux of a broad range of anticancer drugs, the export of glutathione, glutathione conjugates, and glucuronide/sulfate conjugates is primarily mediated by multidrug resistance-associated proteins (MRPs), particularly MRP1. In addition, P-gp expression can be induced by cyclooxygenase-2 (COX-2), a pro-inflammatory enzyme frequently associated with chemoresistance [6].

## 3. Molecular Mechanisms of Ferroptosis

### 3.1. Iron Metabolism and Lipid Peroxidation

Iron represents a critical cofactor in enzymes that drive fundamental physiological processes, including cellular metabolism, oxygen transport, DNA synthesis, and energy production [25]. Beyond its physiological roles, iron serves as a central mediator of ferroptosis by catalyzing ROS generation through Fenton reactions, in which ferrous iron reacts with hydrogen peroxide to produce highly reactive hydroxyl radicals [26]. These radicals promote oxidative damage to lipids, proteins, and DNA, thereby driving cell death under conditions of redox imbalance [27].

Reactive oxygen species (ROS) are critical signaling molecules whose effects are highly concentration-dependent. At physiological levels, ROS act as second messengers that promote cell growth, differentiation, and adaptive stress responses by activating pathways such as MAPK/ERK, PI3K–AKT, and NRF2, thereby maintaining cellular homeostasis. When ROS levels exceed the cell’s antioxidant capacity, they induce oxidative damage to lipids, proteins, and DNA, triggering stress responses that can lead to apoptosis, autophagy, or ferroptosis [28]. In cancer, sustained ROS signaling supports proliferation and survival by inhibiting protein tyrosine phosphatases (PTPs) and sustaining oncogenic cascades, while excessive ROS levels drive lethal oxidative stress.

### 3.2. Ferroptosis in GC

Iron metabolism in GC is profoundly dysregulated. Tumor cells develop a state of “iron addiction,” rewiring their iron uptake, storage, and export pathways to fuel rapid proliferation. This metabolic reprogramming, however, comes at a cost: the same dysregulation expands the labile iron pool (LIP), rendering GC cells particularly vulnerable to ferroptosis, a form of cell death driven by ROS-mediated lipid peroxidation [29]. The LIP in GC is tightly controlled by several key proteins. Iron import is enhanced through upregulation of the transferrin receptor 1 (TFR1), which facilitates excessive uptake of Fe^3+^ via receptor-mediated endocytosis [30]. At the same time, iron export is limited: ferroportin (FPN), the sole known iron exporter, is often downregulated or inhibited by elevated hepcidin levels, preventing iron efflux and promoting intracellular accumulation. Tumor cells also increase ferritin synthesis to store excess iron safely, yet this reservoir can be mobilized through ferritinophagy, a selective autophagic process mediated by the nuclear receptor coactivator 4 (NCOA4), which releases free Fe^2+^ back into the LIP [31]. The consequences of iron accumulation are profound. Excess Fe^2+^ catalyzes the Fenton reaction, producing highly reactive hydroxyl radicals that attack polyunsaturated fatty acids (PUFAs) in cellular membranes. This initiates a chain reaction of lipid peroxidation, generating toxic species such as 4-hydroxy-2-nonenal (4-HNE) and malondialdehyde (MDA) [32]. When lipid peroxidation overwhelms the cell’s antioxidant defenses, including the GPX4 system, it triggers ferroptotic cell death. This iron-driven vulnerability offers a promising therapeutic avenue. Compounds such as Polyphyllin I and Erastin can exploit the heightened iron levels or inhibit antioxidant defenses, inducing ferroptosis and potentially overcoming chemotherapy resistance in GC [33]. By targeting these pathways, it may be possible not only to trigger tumor cell death directly but also to sensitize GC cells to conventional treatments, turning a metabolic liability into a strategic advantage [34]. Indeed, dysregulation of iron metabolism is implicated in a range of diseases, including cancer, atherosclerosis, and neurodegenerative diseases [35]. In GC specifically, the interplay of iron importers, exporters, storage proteins, and ferritinophagy regulators finely tunes the labile iron pool, shaping ROS-mediated lipid peroxidation and ultimately determining ferroptotic susceptibility [36,37].

Ferroptosis is not driven by iron alone: lipid metabolism represents an equally critical determinant of cell susceptibility. Enzymes such as acyl-CoA synthetase long-chain family member 4 (ACSL4) and lysophosphatidylcholine acyltransferase 3 (LPCAT3) incorporate polyunsaturated fatty acids (PUFAs) into membrane phospholipids, generating substrates highly prone to peroxidation [27,38]. Conversely, tumor cells can remodel lipid metabolism to limit PUFA availability or enhance membrane repair, thereby resisting ferroptotic death. This interplay between iron accumulation, lipid composition, and redox balance underscores the multi-layered regulation of ferroptosis and highlights potential vulnerabilities in GC [38].

### 3.3. Antioxidant Defense Systems

Ferroptosis is primarily restrained by antioxidant systems that detoxify lipid hydroperoxides. The cystine/glutamate antiporter system Xc^−^, composed of SLC7A11 and SLC3A2, imports cystine required for glutathione (GSH) synthesis. GSH serves as a cofactor for glutathione peroxidase 4 (GPX4), the key enzyme responsible for reducing phospholipid hydroperoxides to non-toxic lipid alcohols. In GC, overexpression of SLC7A11 and GPX4 suppresses ferroptosis and reduces sensitivity to chemotherapy.

**Figure 1:**
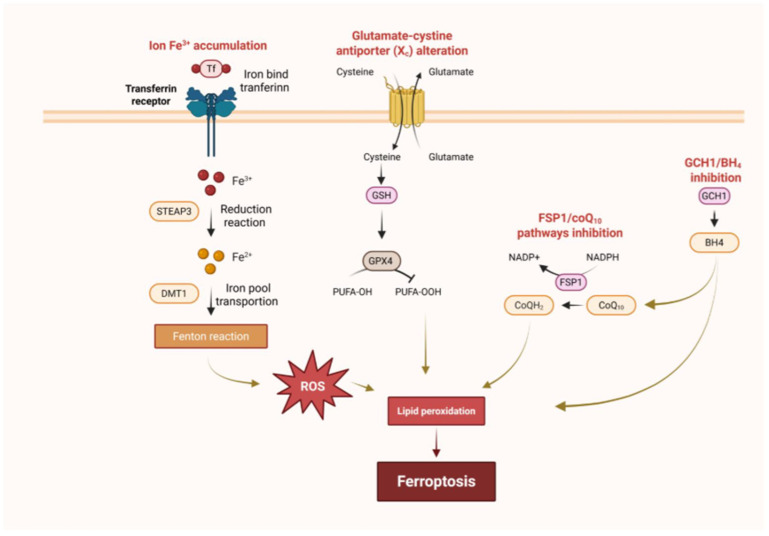
Integrated molecular networks regulating ferroptosis susceptibility. Cellular iron loading via transferrin receptor-mediated Fe^3+^ uptake, subsequent reduction to Fe^2+^ by STEAP3 within the endosome, and DMT1-mediated export into the cytosol, which contribute to the labile iron pool, promote Fenton chemistry and reactive oxygen species (ROS) generation, thereby fueling lipid peroxidation. Concurrent impairment of the System Xc^−^–GSH–GPX4 axis compromises detoxification of phospholipid hydroperoxides. In parallel, inhibition of the FSP1/CoQ10 and cyclohydrolase 1 (GCH1)/BH4 antioxidant pathways attenuates lipid radical scavenging capacity. The coordinated disruption of these redox-regulatory circuits drives unchecked lipid peroxidation and ultimately ferroptotic cell death. Created in BioRender. Calice, G. (2026) https://BioRender.com/x2c4327.

Parallel antioxidant pathways provide redundancy in ferroptosis suppression. These include the ferroptosis suppressor protein 1 (FSP1)–coQ10(coenzyme Q10)–NAD(P)H axis and mitochondrial dihydroorotate dehydrogenase (DHODH), which independently limit lipid peroxidation. The coexistence of these systems highlights the complexity of therapeutically targeting ferroptosis in cancer. The integrated regulatory networks governing iron metabolism, lipid peroxidation, and antioxidant defense in ferroptosis are summarized in Figure 1. Indeed, as illustrated in Figure 1, activation of the SLC7A11–GSH–GPX4 axis enables GC cells to detoxify lipid peroxides and survive chemotherapy-induced oxidative stress, thereby contributing to therapeutic resistance.

### 3.4. Transcriptional and Post-Translational Regulation

Transcription factors such as NRF2 and STAT3 enhance ferroptosis resistance by upregulating antioxidant and cytoprotective genes, including SLC7A11 and GPX4. NRF2 activation enables cellular adaptation to oxidative stress, whereas sustained STAT3 signaling has been associated with aggressive phenotypes and chemoresistance. The tumor suppressor p53 modulates ferroptosis in a context-dependent manner, notably through transcriptional repression of SLC7A11 [39].

Post-translational mechanisms also contribute to ferroptosis regulation. Deubiquitinases such as Ubiquitin Aldehyde Binding Protein 1 (OTUB1) stabilize GPX4, preserving antioxidant capacity under chemotherapeutic stress, and reinforcing ferroptosis resistance [39].

## 4. Ferroptosis and Therapeutic Resistance in GC

### 4.1. Ferroptosis Suppression as a Core Mechanism of Chemotherapy Resistance

Chemotherapy resistance in GC is a multifactorial process in which suppression of ferroptosis has emerged as a central component. Cytotoxic agents such as cisplatin and 5-fluorouracil induce oxidative stress and lipid peroxidation; however, GC cells frequently adapt by reinforcing antioxidant defenses, thereby preventing ferroptotic execution [27].

Upregulation of the SLC7A11/GSH/GPX4 axis represents a major resistance mechanism. Enhanced cystine uptake sustains glutathione biosynthesis and GPX4 activity, detoxifying lipid peroxides, and preserving membrane integrity. High expression of SLC7A11 or GPX4 correlates with poor prognosis and reduced responsiveness to platinum-based chemotherapy in GC patients [40].

GC cells may also exploit alternative cystine transporters, such as SLC7A9, further reinforcing redox homeostasis. Genetic or pharmacological inhibition of these transporters increases lipid ROS accumulation and restores chemosensitivity, underscoring the redundancy of ferroptosis-suppressive networks [20].

### 4.2. Ferroptosis and Resistance to Targeted Therapies and Radiotherapy

Ferroptosis also contributes to resistance against radiotherapy and targeted therapies. Radiotherapy induces iron-dependent oxidative stress and lipid peroxidation; these processes are intrinsically linked to ferroptosis. GC cells with elevated antioxidant capacity or altered lipid metabolism exhibit reduced radiosensitivity, suggesting that ferroptosis suppression contributes to radio resistance [20].

Ferroptosis inducers have demonstrated synergistic effects with radiotherapy and targeted agents in preclinical models, amplifying lipid peroxidation and overcoming adaptive resistance mechanisms [16].

### 4.3. Ferroptosis, EMT, and Metastatic Resistance

Ferroptosis resistance is closely associated with epithelial–mesenchymal transition (EMT) and metastatic competence [41]. Mesenchymal-like GC cells display increased iron sequestration, altered lipid composition, and elevated expression of ferroptosis suppressors, conferring resistance to oxidative stress and cell death [42]. Natural compounds such as dioscin reverse these phenotypes by downregulating SLC7A11 and GPX4, inducing ferroptosis while suppressing migration and invasion [36,42,43]. These findings position ferroptosis induction as a strategy to counteract both therapeutic resistance and metastatic progression [36,41].

## 5. Targeting Ferroptosis: Therapeutic Strategies

### 5.1. Ferroptosis Inducers: Erastin and RSL3

Erastin inhibits system Xc^−^, leading to cystine deprivation, glutathione depletion, GPX4 inactivation, and accumulation of lethal lipid peroxides. RSL3 directly inhibits GPX4 by covalently binding its active site selenocysteine, inducing ferroptosis independently of cystine availability. Both agents trigger iron-dependent lipid peroxidation reversible by ferroptosis inhibitors, confirming pathway specificity [44].

**Figure 2:**
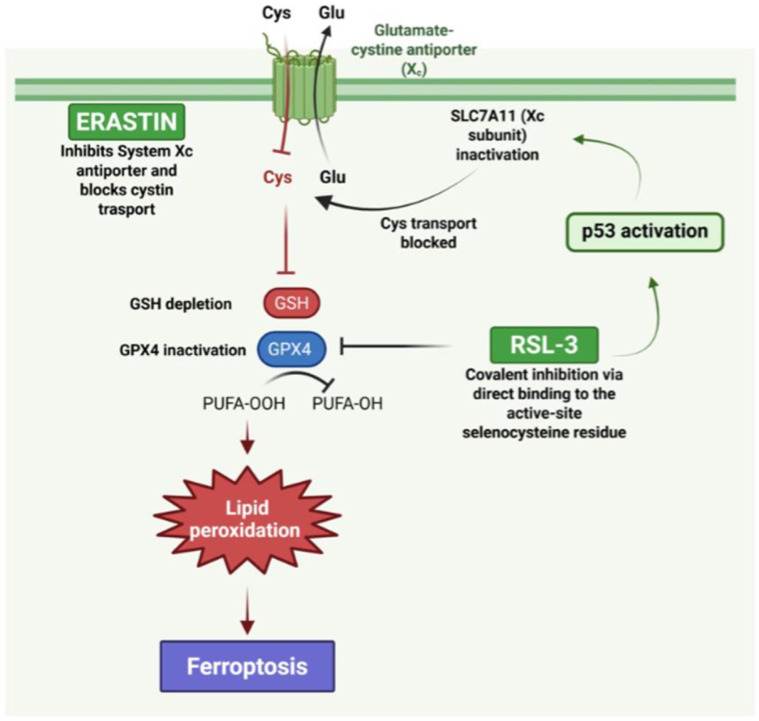
Pharmacologic targeting of System Xc^−^ and GPX4 drives ferroptotic cell death. Erastin inhibits the cystine/glutamate antiporter System Xc^−^ (SLC7A11 subunit), thereby blocking cystine uptake and inducing intracellular glutathione (GSH) depletion. Reduced GSH availability impairs glutathione peroxidase 4 (GPX4) activity, resulting in the accumulation of phospholipid hydroperoxides (PUFA-OOH) and enhanced lipid peroxidation. RSL3 directly inactivates GPX4 through covalent binding to its active site selenocysteine residue. In parallel, p53 activation suppresses SLC7A11 expression, further limiting cystine import. The convergence of these mechanisms culminates in catastrophic lipid peroxidation and execution of ferroptosis. Created in BioRender. Calice, G. (2026) https://BioRender.com/kd1zgc0.

Despite their efficacy, GC cells frequently develop adaptive resistance mediated by NRF2-driven antioxidant programs, including upregulation of FSP1 and aldo-keto reductases (AKRs). Among these, AKR1B1 suppresses ferroptosis by reducing ROS accumulation, increasing GSH levels, and modulating GPX4, ACSL4, and Prostaglandin-Endoperoxide Synthase 2 (PTGS2) expression through STAT3 signaling. Targeting the NRF2–AKR1B1 axis may therefore enhance sensitivity to ferroptosis inducers [38]. Indeed, as illustrated in Figure 2, ferroptosis can be pharmacologically induced through the disruption of the cellular antioxidant defense system centered on the System Xc^−^–GSH–GPX4 axis. Inhibition of the cystine/glutamate antiporter System Xc^−^ by erastin reduces cystine uptake, leading to glutathione depletion and consequent impairment of GPX4-mediated detoxification of lipid hydroperoxides. Similarly, RSL3 directly inhibits GPX4 activity through covalent interaction with its catalytic selenocysteine residue. In parallel, p53-mediated repression of SLC7A11 further restricts cystine availability, amplifying oxidative stress. The combined effect of these mechanisms promotes the accumulation of toxic lipid peroxides and ultimately triggers ferroptotic cell death (Figure 2).

### 5.2. Targeting Lipoxygenases to Suppress Ferroptosis

Lipoxygenase (LOX) enzymes catalyze the oxidation of polyunsaturated fatty acids (PUFAs), promoting lipid peroxidation. Vitamin E and its metabolite α-tocopherol hydroquinone function as radical-trapping antioxidants, suppressing LOX-dependent ferroptosis. Pharmacological inhibitors such as PD146176 and zileuton further highlight the therapeutic potential of LOX modulation [45].

### 5.3. ACSL4 Inhibitors

ACSL4 facilitates PUFA incorporation into membranes, promoting ferroptosis. Thiazolidinediones such as rosiglitazone and troglitazone inhibit ACSL4, suppress lipid peroxidation, and protect against ferroptosis-related tissue injury, highlighting their potential repurposing in cancer therapy [46,47].

### 5.4. Biomarker Development and Combination Approaches

Expression levels of SLC7A11, GPX4, ACSL4, LOXs, and lipid peroxidation signatures represent promising biomarkers for patient stratification. Combination strategies integrating ferroptosis inducers with chemotherapy, radiotherapy, or targeted agents may overcome resistance and improve therapeutic efficacy [48].

## 6. Discussion

The recognition of ferroptosis as a determinant of therapeutic response has substantially expanded current models of drug resistance in GC, moving beyond apoptosis-centered paradigms toward a broader metabolic perspective [48]. Ferroptosis emphasizes the centrality of iron metabolism, lipid remodeling, and redox homeostasis in shaping tumor cell fate under therapeutic pressure [48]. In GC, resistance is increasingly understood as a product of metabolic plasticity, whereby tumor cells dynamically reprogram antioxidant defenses and iron-handling pathways to survive oxidative stress induced by chemotherapy and radiotherapy [20,49,50]. Importantly, ferroptosis suppression does not merely confer drug tolerance; it may also contribute to aggressive biological behavior. By maintaining glutathione-dependent detoxification systems and limiting lipid peroxidation, GC cells preserve membrane integrity and bioenergetic stability, enabling sustained proliferation and survival [41,42]. Emerging evidence further links ferroptosis resistance with epithelial–mesenchymal transition (EMT) [43], stemness traits, and metastatic competence, suggesting that ferroptotic vulnerability may inversely correlate with invasive potential, although these associations remain incompletely characterized in GC and are largely supported by preclinical studies. Therefore, the proposed relationship between ferroptotic vulnerability and invasive potential should be considered preliminary and warrants further validation in patient-derived models and clinical datasets. In this context, ferroptosis acts as both a therapeutic checkpoint and a regulator of tumor progression [41]. Iron metabolism represents a crucial node in this network [36]. The expansion of the labile iron pool enhances oxidative liability, yet GC cells buffer this risk through coordinated upregulation of ferritin storage, NRF2-driven antioxidant transcriptional programs, and GPX4-mediated lipid repair. This delicate balance between iron-fueled proliferation and ferroptosis avoidance underscores the dualistic role of iron as both a driver of malignancy and a potential therapeutic Achilles’ heel. Targeting iron mobilization or ferritinophagy may therefore sensitize tumor cells to ferroptotic death while limiting adaptive resistance [38,51]. Lipid metabolic reprogramming further refines ferroptotic thresholds. Alterations in polyunsaturated fatty acid incorporation into phospholipids, modulation of ACSL4 activity, and shifts in membrane composition collectively determine susceptibility to peroxidative damage. GC cells capable of remodeling membrane lipid composition toward less peroxidizable species exhibit enhanced resistance, reinforcing the concept that ferroptosis is governed by an integrated metabolic circuitry rather than isolated molecular events [47].

The tumor microenvironment (TME) adds an additional layer of complexity. Hypoxia, inflammatory cytokines, nutrient competition, and metabolic crosstalk with cancer-associated fibroblasts and immune cells influence intracellular redox states and iron distribution. Hypoxic signaling can enhance antioxidant pathways and reduce ferroptotic sensitivity, while immune-mediated oxidative bursts may promote lipid peroxidation [44,45]. These spatially and temporally dynamic interactions highlight the necessity of contextualizing ferroptosis within a broader tumor ecosystem. Therapeutic strategies that fail to account for microenvironmental modulation may yield incomplete or transient responses [44].

Importantly, increasing evidence supporting the role of ferroptosis in gastric cancer derives from GC-specific experimental models rather than solely from pan-cancer studies. Investigations performed in GC cell lines, including AGS, MKN45, HGC-27, BGC-823, and SGC-7901, have identified key regulators such as NRF2, SLC7A11, GPX4, ATF3, circHIPK3, and FAM120A as critical determinants of ferroptosis sensitivity and chemotherapeutic response [48]. Furthermore, several of these mechanisms have been validated in xenograft, humanized mouse, and patient-derived xenograft (PDX) models, supporting their biological and translational relevance. More recently, patient-derived gastric cancer organoids have emerged as promising platforms for investigating ferroptosis regulation while preserving tumor heterogeneity and clinically relevant treatment responses. These advanced models may facilitate biomarker discovery, improve patient stratification, and accelerate the development of personalized ferroptosis-based therapeutic strategies [52]. Although preclinical studies consistently demonstrate synergistic effects between ferroptosis inducers and conventional therapies, several translational barriers remain. Redundant antioxidant systems—including FSP1-dependent pathways and mitochondrial protective mechanisms—can compensate for GPX4 inhibition, attenuating therapeutic efficacy [53,54]. Moreover, systemic induction of ferroptosis raises concerns regarding toxicity in normal tissues, particularly those characterized by high iron turnover or oxidative activity. Careful dose optimization, tumor-selective delivery systems, and biomarker-guided patient selection will therefore be critical for safe clinical implementation. The identification of predictive biomarkers represents a central priority. Quantitative assessment of SLC7A11, GPX4, ACSL4, NRF2 targets, iron transporters, and lipid peroxidation signatures may enable stratification of patients according to ferroptotic vulnerability. Integrative multi-omics profiling—combining transcriptomic, metabolomic, lipidomic, and spatial analyses—offers a promising avenue to map ferroptosis-regulatory networks within heterogeneous tumor regions. Such approaches could facilitate the development of precision-based combination regimens that strategically collapse antioxidant defenses while amplifying oxidative stress [39].

Collectively, these insights position ferroptosis at the intersection of metabolism, redox biology, and therapeutic resistance in GC. Rather than functioning as an isolated death pathway, ferroptosis represents a systems-level determinant of tumor adaptability. An additional aspect that deserves further investigation is whether ferroptosis susceptibility differs among molecular GC subtypes. Given the distinct genomic, metabolic, and immune characteristics of chromosomal instability (CIN), genomically stable (GS), microsatellite instability-high (MSI-H), and Epstein–Barr virus (EBV)-positive tumors, ferroptosis-related vulnerabilities may not be uniformly distributed across GC. Future studies integrating molecular classification with ferroptosis biomarkers may help identify subtype-specific therapeutic opportunities [55,56]. Exploiting this vulnerability will require coordinated targeting of iron handling, lipid metabolism, and transcriptional stress responses, ideally integrated with existing cytotoxic and targeted therapies [19,26,48].

## 7. Conclusions

Ferroptosis reframes therapeutic resistance in gastric cancer as a metabolically driven process rooted in redox adaptation and iron-dependent vulnerability. Rather than representing an isolated cell death pathway, ferroptosis integrates metabolic reprogramming, oxidative stress tolerance, and tumor aggressiveness into a unified resistance network. Therapeutic strategies that destabilize this network may shift the balance from adaptive survival to irreversible oxidative collapse. Future clinical translation will depend on biomarker-guided patient selection and rational combinatorial regimens capable of selectively amplifying ferroptotic pressure within the tumor ecosystem.

## Data Availability

All the data generated or analyzed during this study are included in this published article.
